# Sea water temperature and light intensity at high-Arctic subtidal shallows – 16 years perspective

**DOI:** 10.1038/s41597-024-03054-0

**Published:** 2024-02-22

**Authors:** Bernabé Moreno, Anna Sowa, Kamil Reginia, Piotr Balazy, Maciej Chelchowski, Marta Ronowicz, Piotr Kuklinski

**Affiliations:** Marine Ecology Department, Institute of Oceanology Polish Academy of Sciences, Sopot, Poland

**Keywords:** Phenology, Ecosystem ecology, Ecological modelling

## Abstract

Manifestations of climate change in the Arctic include an increase in water temperatures and massive loss of sea ice enabling more light penetration. Yet to understand tempo and scale of these parameters change over time, constant monitoring is needed. We present 16-yr long-term datasets of sea water temperature and relative light intensity at two depth strata (8 and 14 ± 1 m) of two hard-bottom sites in southern Isfjorden proper (Spitsbergen, 78°N). The high temporal resolution of the datasets (every 30 min, between 2006–2022) makes them suitable for studying changes at a local scale, correlating environmental variability with observed processes in benthic assemblages, and serving as ground-truth for comparison with, for example, remotely sensed or mooring data. These datasets serve as baseline for long-term investigations in the shallows of a high-Arctic fjord undergoing severe environmental changes.

## Background & Summary

**Fig. 1:**
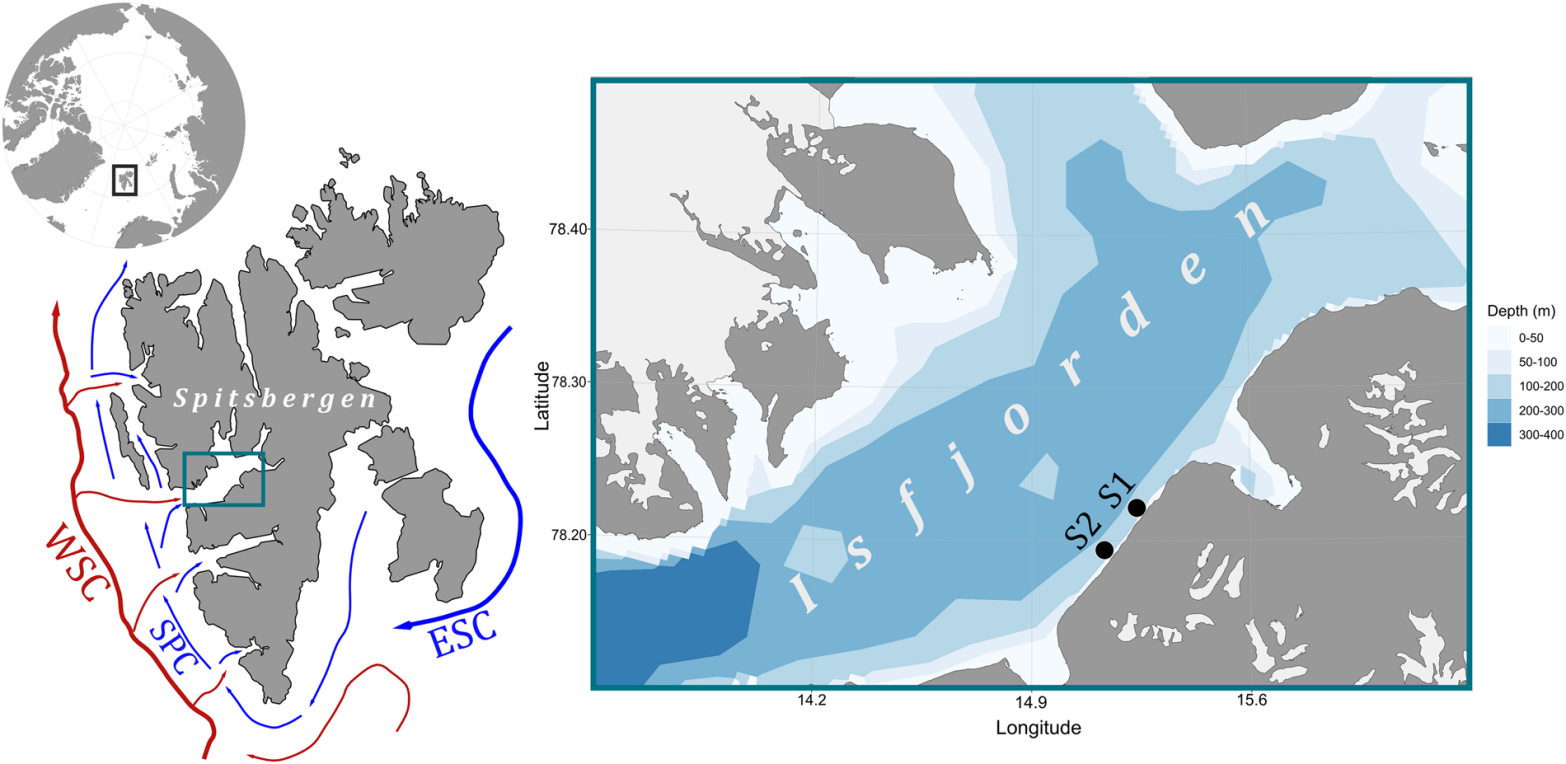
Location of the deployment sites of the temperature and light-intensity loggers. Arctic region in polar stereographic projection using 65°N-limits (top-left); Spitsbergen island and depiction of trajectories of the principal ocean currents: West (WSC) and East (ESC) Spitsbergen Current, and Spitsbergen Polar Current (SPC); and location of S1 and S2 diving sites in southern Isfjorden proper (right). Maps were created using the “PlotSvalbard” R package (https://github.com/MikkoVihtakari/PlotSvalbard).

Warming almost four times faster than the rest of the planet, the Arctic shows one of the most evident responses to global climate change^1,2^ increasingly impacting its relatively young and open for expansion cold-water biotopes^3^. The temperature anomalies observed today are predicted to intensify throughout the upcoming decades with cascading consequences on the system functioning^4^. Light regime in polar regions is highly seasonal. Between 1994 and 2017 the Arctic lost 7.6 × 10^12^ t of sea ice^5^ contributing to greater absorption of incoming solar radiation^6^. The interplay between sea water temperature and light availability causes further changes in heat flux that will have consequences on primary producers within the sea ice (sympagic algae), in the underlying ocean (phytoplankton), and on the seabed (phytobenthos)^7^. Manifestations of the ongoing changes have led to considering the environmental setting of this polar region as the ‘new Arctic’^6^. The iconic high-Arctic Spitsbergen island within the Svalbard archipelago is under the influence of the warm West Spitsbergen Current (WSC), and the cold East Spitsbergen Current (ESC) that continues as the Spitsbergen Polar Current (SPC) west of Svalbard^8^ (Fig. 1). Further, the seawater temperatures and ice conditions around the island in recent years are similar to what is expected for most arctic coastal areas in the second half of this century thus making it a unique study model system and a key transitional region for climate change research^9^. Not surprisingly it is one of the most studied areas in polar research with a substantial body of literature being developed. Nevertheless, continuous sampling and monitoring is logistically complicated especially during wintertime when there is a period of total darkness when the sun is below the horizon for nearly four months (polar night)^10^.

Bulk sea surface temperature (bulk SST, measured between a few centimetres to 5 m below the surface) is one of the most fundamental ocean variables conventionally measured either by research vessels or buoys^11,12^. Continuous data-logging of temperature along the water column is a common practice in oceanography achieved by the installation of moorings, but these are often restricted to deeper (>30 m) and open waters due to navigation restrictions or research interests. Light intensity is a quantitative characteristic of light measured through different approaches among researchers. It can be expressed as luminous flux onto a surface using lumen (lm) per square metre (equivalent to lux; 1 lx = 1 lm m^−2^), watts per square metre (W m^−2^); or adding time (per second) as in micromoles photons (μmol m^−2^ s^−1^) and micro-Einsteins (μE m^−2^ s^−1^). However, knowing the light source is important to convert from lux to other units^13^. More compact data-loggers are a cost-effective alternative that also gives measurements precisely at the study area and in a continuous manner (high temporal resolution), providing valuable environmental information to ecological studies at shallower depths.

Hereby we present sea water temperature (deg C) and light intensity (lux) at sea floor data that were collected as part of a diver-deployed multipurpose experiment assessing the ecology of hard-bottom assemblages in the shallow subtidal of Isfjorden (Fig. 1). Data were recorded to provide environmental information on fundamental parameters to understand their variability. To our knowledge, these are the first sea water temperature and light intensity at sea floor datasets from Isfjorden hard-bottom shallows spanning over fifteen years made publicly available. These datasets are of practical use for studying changes at a local scale, to correlate environmental variability with observed processes in benthic assemblages in Isfjorden shallows or similar fjord systems. This includes, but is not restricted to, studies on light and temperature regimes influencing phytobenthos (e.g., kelps and smaller phototrophs), and light attenuation exerted by drift-ice and darkening of coastal waters. However, the data may be of wider utilisation for ground-truthing, or as a baseline for contrasting remote-sensed data typically of lower spatiotemporal resolution (e.g., satellite imagery focussing on skin SST), extrapolating data from localised moorings to shallow areas within the nearest vicinity, and future comparisons at similar water depths.

## Methods

### Study area

**Fig. 2:**
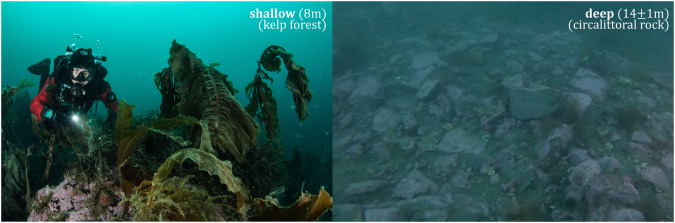
Surroundings of the deployment sites of the temperature and light-intensity loggers. Underwater seascape at the shallow (8 m, left) and deep strata (14 ± 1 m, right) in hard-bottom subtidal. Underwater photographs taken by PK and BM, respectively.

Isfjorden, the largest western Spitsbergen fjord, is subjected to strong seasonality due to its high latitude location (78°N) with glacial meltwater discharge during summer, sea-ice formation during winter, and balancing Atlantic, Arctic, brine- and freshwater inputs^8,14^. The oceanographic conditions in this system are mainly defined by the warmer West Spitsbergen Current (WSC, 3–7 °C conservative temperature Θ, 35.1–35.4 g kg^−1^ absolute salinity S_*A*_), and the Spitsbergen Polar Current (SPC, Θ < 1 °C, 34.5 < S_*A*_ < 35 g kg^−1^) flowing through the sill-free mouth of the fjord^14,15^ (Fig. 1). The twilight season starts in late October and the sun reaches above the horizon in mid-February. The sea-ice season usually starts between late autumn and the beginning of boreal winter, however, Isfjorden has been predominantly sea-ice-free in winter for the last decade^8,16^. Selected stations S1 and S2 were long-term biological and environmental monitoring study sites, two nautical miles apart from each other (Fig. 1). They were located in southern Isfjorden proper where the tidal amplitude does not exceed 1 m, and tidal current is approximately 5 cm s^−1^ ^8^. The submarine environment around the sampling stations is characterised by high-rugosity hard-substrate with frequent sediment pockets. Mixed kelp forests structured by dabberlocks *Alaria esculenta*, sugar kelp *Saccharina latissima*, and *Laminaria* spp. are present in the shallow strata^17^ until ~12 m depth (*pers. obs*.). Sea water temperature and light-intensity loggers placed within underwater forest understories at the shallower stratum were subjected to light attenuation (canopy shading) or partial cover (hydrodynamic swept of kelp blades) compared to the more stable readings at the circalittoral rocks (rocky barrens) found in the deeper stratum (Fig. 2).

### Logging devices, deployment, and recovery

Two different device models were used during the study period. HOBO water temperature Pro v2 (U22-001) (temperature-only) data loggers were deployed during the first year (y_0_, August 2006), and later, HOBO Pendant Temperature/Light 64 K (UA-002-64) data loggers were used until the end of the study (y_16_, July 2022) (Table 1). The UA-002-64 logger senses the light intensity (in lux) of wavelengths from 200–1200 nm (visible light: 400–700 nm), with a higher response towards the infrared spectrum, therefore, the logger’s readings would not correspond exactly to measurements made with a device having different spectral sensitivity (e.g., PAR sensors). This model is most useful for determining relative changes, rather than absolute values of light intensity. Due to battery autonomy, the loggers were set to a 30-min interval between data points. See Table 1 for more technical specification about data loggers. Data loggers were horizontally fixed to multipurpose metal constructions at each depth and site (Table 2) which were ballasted with rocks found in the surroundings^18^. To ensure data recovery, the loggers were replaced *in situ* every year during summer using open circuit scuba diving following the procedures described in^19^. Actions were logged over a logbook to keep track of changes, events, or potential incidents, and to be used during the data curation. For example, in 2015 divers reported that the deep experimental setup at S2 (S2d) was found overturned further down the slope. This event was dated back approximately to 2015-03-13 by identifying datapoints of 0 lux while the rest of the loggers recorded positive values. Temperature measurements, however, were not affected. During that fieldwork campaign (2015-08-06), the construction was restored to its original site at 13 m, but it slipped down again in 2016, then, it was relocated down the slope to less inclined and more stable 15 m (Fig. 2). This latter event was also reported in the logbook, however, unlike the initial one, no precise date was able to be traced back. Therefore, we deemed the light intensity measurements after polar night in 2016 as unreliable until 2016-08-10 when the loggers were exchanged by divers. For simplicity, we will refer to shallow (7–8 m) and deep (14 ± 1 m) constructions.

**Table 1:** Technical specifications and usage period of the data-logging devices.

Data-logger	HOBO Water Temperature Pro v2 (U22-001)^29^	HOBO Pendant Temperature/Light (UA-002-64)^30^	
	Temp	Temp	Light
Operation range in water	−40° to 50 °C	−20° to 50 °C	0 to 320,000 lux
Accuracy	± 0.21 °C	± 0.53 °C	—
Resolution (at 25 °C)	0.02 °C	0.14 °C	—
Stability (drift)	0.1 °C/year	< 0.1 °C/year	—
Waterproof (m)	120	30	
Weight (g)	42	18	
Dimensions (mm)	30 × 114	58 × 33 × 23	
Usage period	2006-08-25/2007-08-16	2007-08-17/2022-07-29

**Table 2:** Position of the data loggers and periods of data gaps.

Station	Depth (m), stratum	Geographical coordinates (N, E)	Missing data period	Number of months with missing data
S1	8 m, shallow	78.21292, 15.23556	2013-11-6/2014-08-15	9
S1	13 m, deep	78.21303, 15.23389	2014-03-25/2014-08-15	5
S2	7 m, shallow	78.1883, 15.1447	2010-07-20/2011-08-26	1
			2014-03-18/2014-08-15	5
S2	15 m, deep	78.1879, 15.1451	2014-01-18/2014-08-15	7
			2020-07-26/2021-07-31	12

## Data Records

### Data curation

The data were curated and assembled removing any measurements taken above seawater surface. As specified by the ACDD Attribute Convention for Data Discovery v.1.3^20^, date and time in the CF-NetCDF (.nc) files are made available following the ISO 8601:2004 recommended timestamp, hour information is provided in a 24-hour format and standardised to UTC: “YYYY-MM-DDThh:mm:ssZ”. During recovery operations, mobile epibenthic organisms such as gastropods, chitons, and echinoids were occasionally observed on top of the light sensor. In the shallow stratum, random movement of kelp blades could also periodically obstruct the sensor which takes a measurement only directly above it. Therefore, data points recording 0 lux during periods of light are thought to be a consequence of these incidences. Other instances of false 0 lux readings were noted in 2015 and 2016 at S2 deep (S2d) presumably as the result of the constructions becoming unstable and falling down the slope. During both of those logging periods the temperature measurements were unaffected. On the other hand, there was an instance of a non-natural light intensity reading (>2500 lux) during the polar night caused by the firing of artificial light source of scientific divers (photography flashes, or strobe lights used for orientation underwater^19^). This datapoint was corrected to zero, in agreement with the neighbouring datapoints. Data gaps are attributed to the overall malfunction of the loggers or premature battery exhaustion. Those gaps in readings of both parameters occurred in all stations and depths (Table 2). An unknown period of no light intensity measurements occurred at S1 shallow (S1s) between 2018-09-02 and 2019-07-30 which we assume to be a result of light intensity sensor malfunction as no other incidents were reported and the temperature was logged continuously throughout the time of deployment.

The curated merged file together with processed subfiles including monthly and annual means are accessible in .csv format through the Figshare repository^21^. NetCDF files (.nc) and corresponding metadata following the NetCDF Climate and Forecast (CF) metadata conventions are available separately for S1s, S1d, S2s, and S2d through IOPAN Geonetwork^22–25^. All available files are briefly described in Table 3.

**Table 3:** Details and description of the dataset publicly available via Figshare^21^ (13 .csv, 1 .xlsx, 4 .nc files; total size 108.35 MB), and IOPAN Geonetwork repositories^22–25^ (4. nc files; total size 11.98 MB).

Filename	Description	Size	Number of data points (records)
Isfjorden_temp-lux_seafloor_2006-2022_S1_8m-S1s.nc	Sea water temperature and light intensity at sea floor data (2006–2022) at station S1, 8m (S1s)	3.05 MB	264355
Isfjorden_temp-lux_seafloor_2006-2022_S1_13m-S1d.nc	Sea water temperature and light intensity at sea floor data (2006–2022) at station S1, 13m (S1d)	3.13 MB	271013
Isfjorden_temp-lux_seafloor_2006-2022_S2_7m-S2s.nc	Sea water temperature and light intensity at sea floor data (2006–2022) at station S2, 7m (S2s), Isfjorden (78°N)	2.91 MB	252138
Isfjorden_temp-lux_seafloor_2006-2022_S2_15m-S2d.nc	Sea water temperature and light intensity at sea floor data (2006–2022) at station S2, 15m (S2d)	2.89 MB	250357
S1s.csv	Sea water temperature and light intensity at sea floor at 8 m in S1.	12.42 MB	264355
S1d.csv	Sea water temperature and light intensity at sea floor at 13 m in S1.	12.76 MB	271013
S2s.csv	Sea water temperature and light intensity at sea floor at 7 m in S2.	11.38 MB	252138
S2d.csv	Sea water temperature and light intensity at sea floor at 15 m in S2.	11.32 MB	250357
globalAttributes_all.xlsx	Xlsx file containing the global attributes used to encode the NetCDF files. One sheet per site-depth.	18.44 kB	—
mergedFiles_Isfjorden.csv	Merged file containing curated temperature and light intensity data from all sites-depths (S1, S1d, S2s, S2d)	48.45 MB	1037863
monthlyMean_S1shallow.csv, monthlyMean_S1deep.csv, monthlyMean_S2shallow.csv, monthlyMean_S2deep.csv	Monthly averages, maxima, and minima, of sea water temperature and light intensity for each station/depth.	6.30 kB 6.75 kB 6.31 kB 6.25 kB	184, 188, 176, 175
annualMean_S1shallow.csv, annualMean_S1deep.csv, annualMean_S2shallow.csv, annualMean_S2deep.csv	Annual averages, maxima, and minima, of sea water temperature and light intensity for each station/depth.	631 B 630 B 636 B 634 B	17 each

## Technical Validation

Similar data loggers and deployment approach have been used to gather environmental background information in studies on colonisation^26^ and succession^27^ of hard-bottom assemblages, and ecological structure and carbon standing stock within kelp forests^28^, proving its wide utility in ecological investigations. Light intensity values reported in our dataset fall within the ranges reported by research conducted at similar sublittoral depths, and contrasting environments such as kelp forests^27,28^ and barren grounds^27^ in different marine ecoregions.

### Data visualisation

**Fig. 3:**
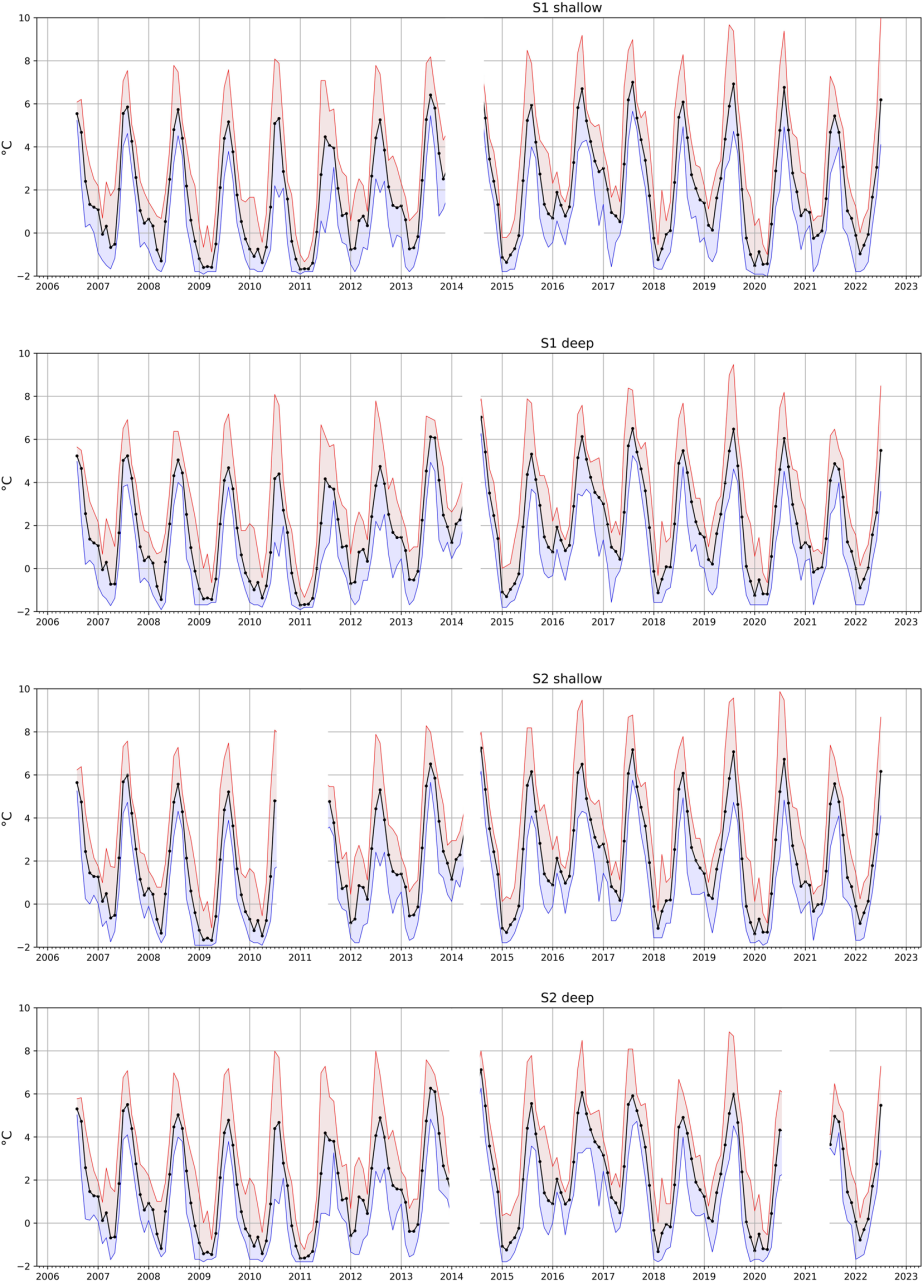
Monthly average (black line), maximum (red), and minimum (blue) temperatures at study sites and depth strata in Isfjorden shallow subtidal. Data gaps in blank (see Table 2).

**Fig. 4:**
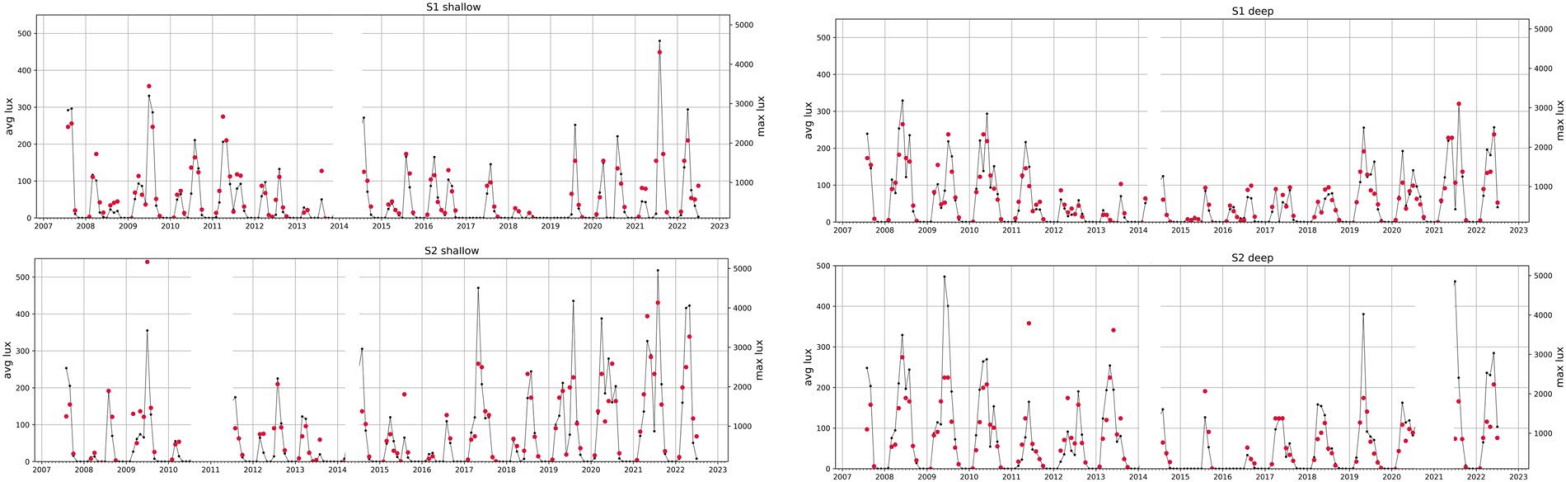
Monthly averages (left axes, black lines) and monthly maxima (right axes, red dots, axes min values set to 100) of light intensity (lux = lum m^−2^) at study sites (S1 top, S2 bottom) and depth strata (shallows left, deeps right) in Isfjorden shallow subtidal. Data gaps in blank (see Table 2).

After curation of the merged file, environmental data were plotted to observe trends, possible drift, or any other misreading. There was no observed drift, and the sole unintentional error previously mentioned in the data has been rectified. Overall, no increasing trends in the bottom temperature were observed during the study (Fig. 3), suggesting more stable conditions compared to the atmospheric amplification trends^2^. However, evident fluctuations were noted especially during the winter period. Considering the monthly averages over the years the summer temperatures appeared rather stable. It was during the polar winter seasons that readings revealed unexpected fluctuations and periods of increased temperatures in some years. No evident trends could be observed within the light intensity data other than the expected lower values in deep strata and light attenuation towards the winters (Fig. 4). For reasons unable to specify without further analyses, higher values of light intensity were recorded at the S2 study site. Due to the possible influence of mobile fauna and the temporary overlay of kelp blades above the light sensor, it is impossible to form any clear conclusions from the collected data at this stage. Besides the obvious seasonality of the Arctic region, other abiotic aspects that can affect the light incidence come to play such as prominent cloud cover and presence of sea ice^7^.

## Data Availability

The code that accompanies this data descriptor is publicly available in the GitHub repository https://github.com/8ernabemoreno/Isfjorden-shallows_longterm-seawater-temp-lux. It contains Python code that might be useful for basic level users to create CF-NetCDF (.nc) files from .csv, and (ii) minimally process long-term data (e.g., annual, and monthly means).
